# A framework to assess patient-reported adverse outcomes arising during hospitalization

**DOI:** 10.1186/s12913-016-1526-z

**Published:** 2016-08-05

**Authors:** Okoniewska Barbara, Santana Maria Jose, Holroyd-Leduc Jayna, Flemons Ward, O’Beirne Maeve, White Deborah, Ocampo Wrochelle, William A. Ghali, Alan J. Forster

**Affiliations:** 1 W21C Research and Innovation Centre, G-01- TRW Building, 3280 Hospital Drive, NW, Calgary, AB T2N 4Z6 Canada; 2 Department of Community Health Sciences, O’Brien Institute for Public Health, Cumming School of Medicine, University of Calgary, 3rd Floor, 3-36E, TRW Building, 3280 Hospital Drive, NW, Calgary, AB T2N 4Z6 Canada; 3 Department of Medicine, Cumming School of Medicine, University of Calgary, 1403 29 St NW, Calgary, AB T2N 2 T9 Canada; 4 Family Medicine and Primary Care Research Office, University of Calgary, G012, Health Sciences Centre, 3330 Hospital Drive NW, Calgary, Alberta T2N 4 N1 Canada; 5 Faculty of Nursing, University of Calgary, 2500 University Drive NW, Calgary, AB T2N 1 N4 Canada; 6 Department of Medicine, University of Ottawa, Civic Campus 1053 Carling Avenue, Box 684, Ottawa, ON K1Y 4E9 Canada

**Keywords:** Patient-reported adverse outcomes, Patient safety, Medical informatics, Transitions of care, Electronic health records

## Abstract

**Background:**

The assessment of adverse events from a patient-centered view includes patient-reported adverse outcomes. An *adverse outcome* refers to any suboptimal outcome experienced by the patient; when adverse outcomes are identified through a patient interview these are called *patient-reported adverse outcomes*. An *adverse event* is an adverse outcome that is more likely due to the processes of medical care rather than to the mere progression of disease. In the context of a large-scale study assessing post-hospitalization adverse events, we developed a conceptual framework to assess patient-reported adverse outcomes (PRAOs). This methodological manuscript describes this conceptual framework.

**Methods:**

The PRAO framework builds on a validated adverse event ascertainment method including three phases: Phase 1 involves an inquiry to ascertain the occurrence of any patient-reported adverse outcome. It is completed by a structured telephone interview to obtain details – from a patient perspective – on symptoms that developed and/or worsened after hospitalization. Phase 2 involves the classification of PRAOs by physicians not involved in the patient care. Physician-reviewers then rate the PRAOs using well-adopted scales to determine whether the occurrence was the natural progression of the underlying illness or due to medical care. When the PRAO is rated as “due to medical care”, it is then classified as an “adverse event”. Phase 3 involves the classification of adverse events as preventable or ameliorable.

**Results:**

Out of the 1347 patients contacted at 1-month post-discharge, 469 reported AOs and after reviewing 369 cases, 29 were classified as AEs. Observed agreement levels between raters were 87.3, 85.5, and 85.2 % respectively displaying a good agreement (k > 0.60).

**Conclusion:**

The framework incorporates PRAOs as a way to identify cases that need to be evaluated for adverse events. Further validation of this framework is warrant with the final aim of implementation at larger scale. The implementation of this framework will enable clinicians, researchers and healthcare institutions to compare outcome rates across providers and over time.

**Electronic supplementary material:**

The online version of this article (doi:10.1186/s12913-016-1526-z) contains supplementary material, which is available to authorized users.

## Background

Since the late 1990s when the Institute of Medicine released its influential report “To err is human”, there have been increased efforts to enhance patient safety and to quantify the rate of adverse events (AEs) [1]. The reported rate of AEs for in-hospital patients ranges from 2.5 to 7.5 % [2, 3], although recent literature suggests that these rates may be a gross underestimation, with the actual rate ten times greater than previously reported [4]. Two recent North American studies found an incidence of post-hospital discharge AEs ranging from 19 to 23 % [5–7]. The discrepancies in these numbers highlight the lack of a gold standard technique for determining and assessing AEs.

Indeed, various methods exist within the literature for the collection and ascertainment of AEs, including self [8–10] and incidence reporting, conduction of full retrospective patient record reviews, malpractice claims analyses and mortality and morbidity reviews [11, 12]. The problem with these methods is that they are time intensive, resource heavy, often identify different types of AEs and miss injuries occurring in the post discharge period.

There has been a movement within the literature to turn towards patients to help identify adverse events [13–18]. Zhu et al., demonstrated that patients recognize and accurately identify in-hospital adverse events [13]. In addition, Weingart et al. showed that many patient identified events were not captured by traditional incident reporting systems [14]. Furthermore, King et al. [18] discussed how the integration of patients’ perspectives facilitates the understanding of adverse events and suggest the inclusion of patients’ perspective as a complementary measuring tool.

Forster et al. [7] identified that one in five patients experienced an adverse event in the transition from hospital to home, otherwise known as the post discharge period, a period that is missed by the traditional methods of adverse event ascertainment. These studies show the necessity of involving patients to improve patient safety.

Forster et al. [7] developed a method where the patient identified potential adverse events. Patients were contacted within 2–5 weeks after discharged and were asked to identify any new or worsening conditions, or otherwise known as a patient reported adverse outcomes (PRAO), as well as health services utilized. Patients who identified a PRAO had a case summary prepared that was reviewed by two board certified internists, to determine if an adverse event occurred [7]. In a follow up study, the authors assessed the reliability of the peer review process for the ascertainment of adverse events and concluded that for the best agreement, at least three similarly-trained reviewers are required [16].

This methodological paper describes the framework developed for the assessment of patient-reported adverse outcomes (PRAOs) occurring at the transition between the hospital and the community. The framework is an extension of the method developed by Forster et al. This modified method substitutes the medical chart review, for the review of patient cases that include clinical information from discharge summaries and the patient reports [6, 7, 16, 19–21].

## Methods

The present study was nested in a large-scale randomized clinical trial (RCT). This RCT assessed the efficacy of a structured web-accessible electronic discharge summary being compared to usual care discharge summary dictations [22, 23]. The full details of this RCT has been described elsewhere [22]. This study uses data collected during the RCT.

In this section we present the multi-phased method included in the framework overview.

### Framework overview

The conceptual framework for assessing PRAOs was developed to aid in the understanding of concepts and to act as a guide for future researchers conducting similar research [24]. Using an iterative process, we made modifications to the approach designed by Forster et al. [6, 7, 16, 20, 21] to enable the assessment of PRAO that could be performed using hospital discharge summaries. Figure 2 displays the framework, and its three phases of information including, Phase 1 describes the identification of PRAOs; Phase 2 includes two steps: 1) evaluating causation and severity of the PRAO. When the PRAO is likely to be related to medical care (omission or commission) then the PRAO is considered an adverse event; 2) using a modified 6-point scale to assess whether the AE was due to treatment/suboptimal medical care. [16]; Phase 3 classifies the identified AE as ameliorable, preventable or neither.

We have clarified the terminology used to describe the conceptual framework (Table 1).

**Table 1:** Definition of terms

Term	Definition
Adverse Outcome (AO)	Any suboptimal outcome experienced by the patient, including a new or worsening symptom, unanticipated visits to health facilities or death
Patient-reported Adverse Outcome (PRAO)	Any suboptimal outcome experienced by the patient and reported directly by the patient without interpretation of anyone else, which includes a new or worsening symptom, unanticipated visits to health facilities or death
Adverse Event (AE)	An adverse outcome caused by the processes of medical care rather than by the progression of disease, where medical care refers to all aspects of care
Ameliorable Adverse Event (AAE)	An injury whose severity could have been *substantially reduced* if different actions or procedures had been performed or followed (unavoidable injuries but severity could have been decreased)
Preventable Adverse Event (PAE)	An injury that could have been *avoided*

**Fig. 1:**
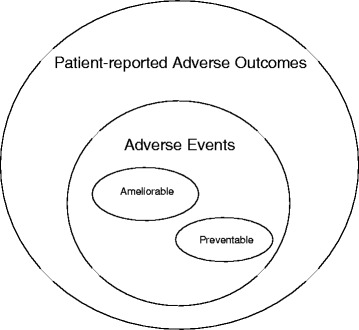
Schematic representation of how the concepts described and assessed in the paper relate to one another

The most important terms underlying the framework are: *adverse outcomes*, *patient-reported adverse outcomes (PRAO)*, *adverse events (AE)*, *ameliorability,* and *preventability*. Figure 1 is a schematic representation of how the concepts relate to one another. An *adverse outcome* is defined as any suboptimal outcome experienced by the patient, including a new or worsening symptom, an unanticipated visit to a health facility, or death [6, 7]. Adverse outcomes can be identified by medical record review or through a patient interview. If captured only through the latter, we define the outcomes as “*patient reported adverse outcomes*” or PRAOs. An *adverse event* is an adverse outcome that is more likely due to the processes of medical care rather than by the mere progression of disease, where medical care refers to all aspects of care including the commission or omission of required acts [6, 7]. An *ameliorable adverse event* is an outcome whose severity could have been substantially reduced if different actions or procedures had been performed or followed [6, 7]. A *preventable adverse event,* meanwhile*,* is an outcome that could have been potentially avoided.

#### Phase 1: PRAO identification

In our study [22, 23], PRAO identification occurs at one month post-hospitalization [24]. A trained interviewer administers a brief structured interview over the telephone that takes approximately ten minutes to complete. The questionnaires (see Additional files 1, 2 and 3) gives the patient an opportunity to describe new or worsening health conditions as symptoms. We ask patients to describe: 1) timing of the onset of symptoms; 2) duration of symptoms; 3) severity of the symptoms (on a 10-point scale; 1 = not bothersome; 10 = bother a great deal). Patients also explain the actions taken to alleviate the symptoms, including their use of health services and report if and how the symptom limited their usual physical activities. The PRAO data generates a narrative interview summary. This summary is used in subsequent phases.

#### Phase 2: adverse event determination

The narrative interview summary is combined with information obtained from the electronic medical record (specifically, the transfer-of-care summaries, otherwise known as discharge summaries, related to the index hospital encounter and subsequent visits) [6, 7, 20] generating a case report. This case report is used by the physician raters to determine the cause of the adverse event. Two physicians review the report independently to determine whether the cause of the outcome was due to the natural progression of the patient’s health condition or to the medical care received. Our team of reviewers consists of three academic physicians; two Royal College certified internists and one College-certified family physician.

During this phase, the physicians perform their classification of causation based on a simple dichotomous choice (i.e. the outcome was likely caused by medical care or not). If the PRAO is rated by the reviewing physician as being likely to have been caused by medical care, it is then classified as an adverse event. Once a PRAO is classified as an AE, the physician reviewers rate their type. Type includes the following groups: adverse drug events, procedure-related injuries, nosocomial infections, care related falls, therapeutic errors, diagnostic errors, and other. Severity is measured in terms of the impact on the patient (how it affected the patient and how the patients felt and functioned) and in terms of the impact on health services used (e.g. visit to the emergency department).

Recognizing that a dichotomous assessment of relation of a PRAO to medical care may be difficult, we also presented reviewers with a more nuanced 6-point scale for judging the link of a PRAO to quality of medical care. The 6-point scale’s response options are:

1. No evidence that outcome was due to treatment and/or suboptimal medical care;
2. Little evidence that outcome was due to treatment and/or suboptimal medical care;
3. Outcome was possibly due to treatment and/or suboptimal medical care (50/50 chance) but was more likely due to disease;
4. Outcome was possibly due to treatment and/or suboptimal medical care (50/50 chance) and was more likely due to treatment and/or medical care than disease;
5. Outcome was probably due to treatment and/or suboptimal medical care;
6. Outcome was definitely due to treatment and/or suboptimal medical care.

#### Phase 3: preventability and ameliorability determinations

We then brought forward any cases with a rating of ≥5 on the above-mentioned 6-point scale to phase 3 for a final evaluation step using Additional file 4. In this final step, the physician reviewers were asked to classify whether the adverse event is preventable, ameliorable or neither, with definitions of these constructs as outlined earlier.

#### Ascertainment of agreement

Disagreements among reviewers can occur during phases 2 and 3 of this multi-phased process. In phase 2, when a disagreement between raters occurs, the raters can meet face-to-face to come to a consensus. If a disagreement persists, a third rater can act as a tiebreaker.

When using the 6-point scale as the primary tool for determining link of a PRAO to medical care, reviewers similarly need to review and discuss cases when their ratings are disparate. This is needed when one physician rates ≥5, while the other physician rates ≤3. If one physician rates ≥5 and the other rates 4, the higher rating is endorsed and this is considered an AE. Similarly, when one physician rates ≤3 while the other rates 4, the lower rating is endorsed and this is not considered an AE.

## Results

All patients admitted to a medical teaching unit in a Canadian tertiary hospital were invited to participate in the study [22]. During a period of 18 months, 1347 patients were interviewed to report any adverse outcome that rose at one month after discharge from hospital. Of these patients 469 reported adverse outcomes, 369 cases were reviewed of which 29 were rated as adverse events by the raters with an overall adverse event rate of 7.9 %. Out of these, 11.5 % were preventable, 69.3 % were ameliorable and 19.2 % were classified as neither preventable nor ameliorable.

All 369 cases were reviewed by at least 2 reviewers. Reviewers 1, 2, and 3 each reviewed 266, 199, and 273 cases, respectively. Reviewers 1 and 2 rated a total of 96 cases between themselves; reviewers 2 and 3 rated 103 cases between themselves; and reviewers 1 and 3 rated a total of 170 cases between themselves. Table 2 shows the phase 2 agreements between raters prior to any re-rating efforts, with observed agreement levels of 65.3, 75.2, and 52.4 % respectively, and corresponding kappa (k) values of 0.30, 0.33 and 0.14, respectively. These kappa levels indicate only slight to fair agreement [25]. After re-rating, the observed agreement levels were 87.3, 85.5, and 85.2 % respectively, and corresponding kappa (k) values of 0.62, 0.76 and 0.67 (*p* < 0,001) [25].

**Table 2:** Phase 2 agreements between reviewers *prior* to re-rating on the dichotomous choice of whether the outcome was due to medical management or not. (*N* = 369)

	Agreement (%)	Kappa (k)	P-value
Reviewer 1 and 2	65.3	0.30	0.004
Reviewer 2 and 3	75.2	0.33	0.001
Reviewer 1 and 3	52.4	0.14	0.013

**Fig. 2:**
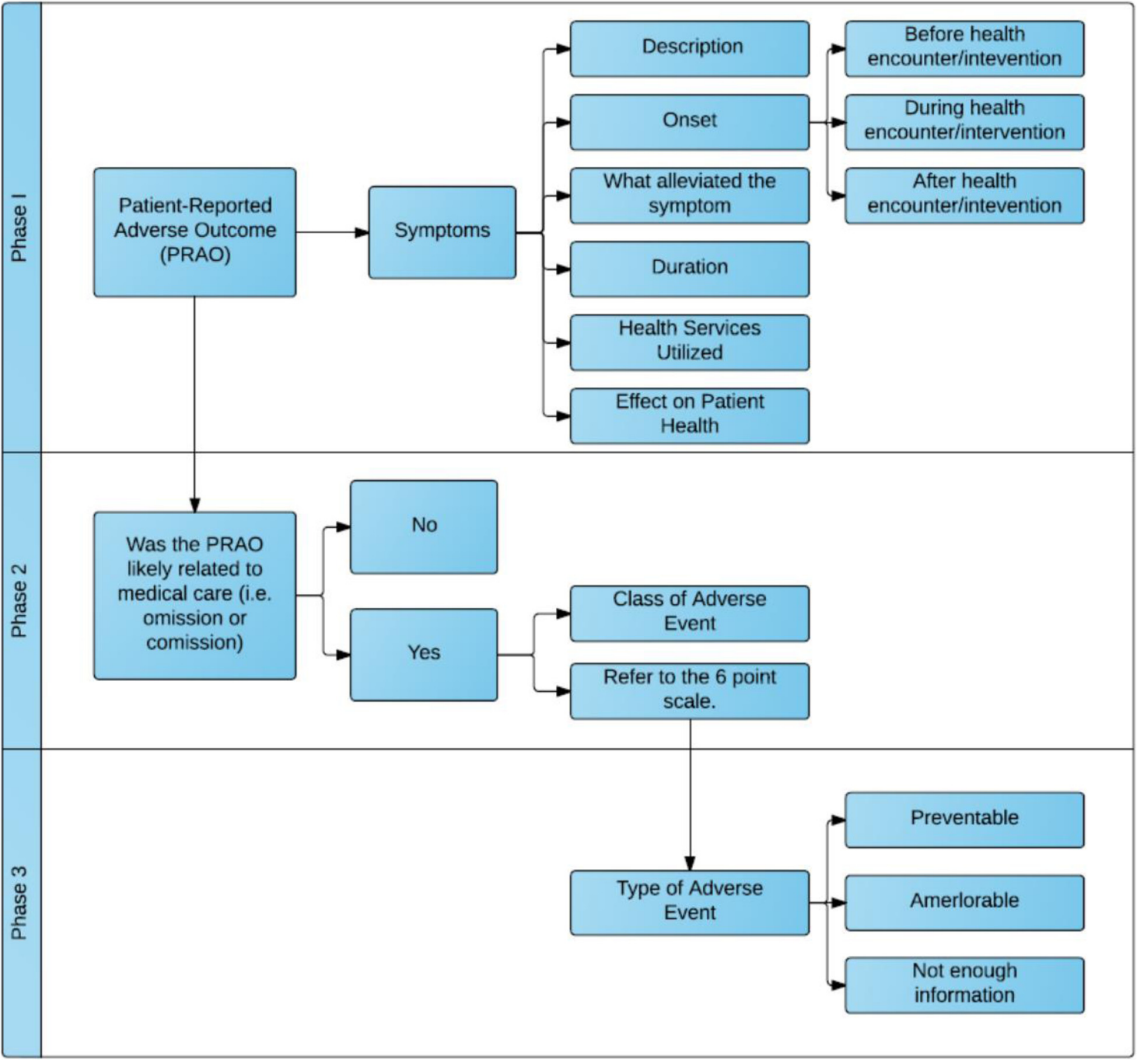
Flow chart describing the evaluation process of a patient-reported adverse outcome (PRAO)

Table 3 shows the agreement between reviewers after re-rating occurred using the 6-point scale of the PRAO framework (Fig. 2). Reviewers 1 and 2 had a 94.8 % agreement with k = 0.82 (*p* = 0.001). Reviewers 2 and 3 had a 91.3 % agreement, k = 0.81 (*p* = 0.001) and reviewers 1 and 3 had a 91.8 % agreement, k = 0.74 (*p* = 0.001). There were 21 cases that reviewers had full agreement on after re-rating as being classified an AE due to medical care (i.e. both reviewers rated the PRAO between a 5 and 6), which indicates an initial adverse event rate of 5.7 %.

**Table 3:** Phase 3 ratings from reviewers using the 6-point scale of the PRAO framework after re-rating

Reviewer 1	Reviewer 2				
		1–2	3	4	5–6
	1–2	37	17	8	0
	3	7	8	1	2
	4	1	0	2	0
	5–6	1	2	3	7
*N* = 96, 94.8 % agreement, k = 0.82, *p* = 0.000					
Reviewer 2	Reviewer 3				
		1–2	3	4	5–6
	1–2	29	8	5	2
	3	12	13	2	6
	4	1	1	6	4
	5–6	0	1	10	3
*N* = 103, 91.3 % agreement, k = 0.81, *p* = 0.000					
Reviewer 1	Reviewer 3				
		1–2	3	4	5–6
	1–2	50	30	31	2
	3	6	11	0	2
	4	0	0	4	4
	5–6	3	8	8	11
*N* = 170, 91.8 % agreement, k = 0.74, *p* = 0.000

There were 30 instances in which reviewers still disagreed after meeting and reassessing cases (i.e. one reviewer rated that PRAO between 1 and 3, while the other rated it between 5 and 6). In such instances, a third reviewer assessed the case and this rating was taken as final (Table 4).

**Table 4:** Number of PRAOs that still resulted in disagreement between two reviewers, which were rated by a third reviewer

	Disagreement (%)	Rated by Third Reviewer (N)
Reviewer 1 and 2	5.2	6
Reviewer 2 and 3	8.7	9
Reviewer 1 and 3	7.6	15^a^

^a^One case that was a disagreement was not re-rated, but a third reviewer rated it instead

To further illustrate the PRAO rating process, we have provided real examples that can be found in Additional file 5 (a, b, c). These examples include full agreement on AE, agreement on not an AE, and an example of disagreement.

## Discussion

In this paper, we have refined and presented a conceptual and methodological framework for the assessment of PRAOs. The framework builds on a validated adverse event ascertainment method, with modifications to streamline the process by including only the discharge summary as well as the patient report [6, 7, 19, 20]. This method identifies PRAOs using a structured interview via the telephone, classifies the cause of the PRAOs and determines if it was due to medical care and whether the PRAO was ameliorable or preventable. The framework presents a potential tool to assess patient safety that can be used by other groups such as healthcare quality improvement management, and with wide spread adoption, it can help standardize the ascertainment of AEs.

The importance of using PRAO in the ascertainment of adverse events is highlighted in several studies [13–18, 26–28]. Traditionally it has been argued that patients lack the expertise to evaluate care received [28]. However, patients’ experiences and perceptions are related to indicators such as access to medical care, patient-provider communication, and transitions of care [9, 27]. Positive patient experiences are linked to patient health status [27], while negative patient experiences have been linked to adverse health outcomes [5]. In addition, patients are aware of adverse events and are often accurate in reporting when adverse events have occurred. It has also been found that patients report events that would have been missed using standard chart review techniques [14, 26, 27, 29, 30]. Utilizing PRAO can be a less costly method for the identification of adverse events as compared to the traditional methods. The novelty of this framework is the incorporation of PRAOs as a way to identify cases that needed to be evaluated for adverse events [7]. To our knowledge, no framework has been developed describing the assessment of adverse events utilizing electronic transfer-of-care discharge summaries and PRAO.

Many different methods exist within the literature for the collection and ascertainment of AEs, but each method is faced with limitations [3, 10, 31–33]. Self-reporting of adverse events has been one common method utilized in institutions, although voluntary reporting often underreports the rate of AEs [10–12, 34]. Conducting full retrospective patient record reviews has been regarded by some groups as the gold standard for the assessment of AEs [33, 35]. This method typically consists of a two-phase chart review process, involving a research nurse and a physician rating adverse events [3, 10, 31–33]. This is an onerous process that requires a significant amount of time and resources, and relies on events having already occurred or a healthcare professional identifying an event [10]. Furthermore, this method does not capture post-hospital AEs and excludes patients’ reports, which we know are invaluable and capture missed events [13, 14].

Adverse event ascertainment is often time and resource intensive, due to amount of documentation required from the chart. In order to streamline the adverse event ascertainment process, our framework proposes the use of hospital discharge summaries as a key source of information for PRAO assessments, supplemented by direct interviews with patients. Previous studies have shown that discharge summaries contain the necessary information required to assess adverse events [36]. When Melton and Hripcsak [37] compared assessments of adverse events using full chart reviews versus discharge summaries, their results showed that the assessments were similar. Furthermore, some studies have shown the potential for the use of automated detection systems that are able to scan discharge summaries for adverse event triggers [36–40]. Electronic transfer-of-care summaries can provide a platform for this technology.

There are four limitations to this method of AE detection. Firstly, patients can report a large variation in the amount of outcomes, not all of which are related to adverse events. Previous studies have shown that approximately 50 % of patients will report an adverse occurrence to an interviewer after being discharged from the hospital, although the actual AE rate was 9 per 100 admissions [14]. This high rate of PRAOs leads to a large volume of cases that need to be evaluated by reviewers, increasing the burden to raters. Faced with this challenge, the use of well-trained research assistants conducting patient follow up calls is a potential solution.

Secondly, as with any retrospective study utilizing an interview technique, there is a potential for recall bias. To minimize recall bias, interviewers provide patients with probes to assist recall along with possible responses that offer patients set time frames around which they can structure their answers [41–44]. For example, for symptom duration, patients can report less than one day, less than one week, less than two weeks, two weeks or more, or still occurring. By proposing a standard process for data collection and review, we hope that the effect of this bias will be minimized. If all institutions and researchers perform this work in the same way, then the impact of this recall bias will be consistent.

Novick suggests that the traditional bias against telephone interviews, low quality of data is not supported by robust evidence [45]. This bias had been attributed to the lack of visual cues during telephone interviews that could potentially result in probing and interpretation of responses. However on the other hand, telephone-interviews may allow respondents to feel relaxed and able to disclose sensitive information [45–47].

Thirdly, a known limitation within the traditional methodology to ascertain AEs is the inter-rater reliability within the point scale [6, 16, 48]. It has been our observation that different raters have varying exposures to AEs resulting in each individual viewing a case differently. It is for this reason that we have suggested incorporating an “all-or-none” phenomenon, which leaves little room for disagreement. However, if a point scale is used, we suggest that reviewers are carefully chosen to have similar training, exposure to AEs, are from the same institution and that regular and frequent meetings are conducted to ensure all reviewers have a firm understanding of the definitions associated with AEs and what exactly is being rated. Furthermore, we suggest that reviewers undergo a training program similar to that used by Brennan and colleagues to help improve inter-rater reliability [49]. While there is debate within the literature concerning the reliability of using one rater versus two or more, most studies suggest that more than one reviewer is required to agree on a case before the event can be classified as an AE [15, 48]. These are all factors that can be modified as our knowledge of optimal rating procedures improves.

Finally, we found in our study that in some instances reviewers had difficulty deciding between a 3 (the outcome was *possibly* due to treatment and/or suboptimal medical care (50/50 chance) but was *likely* due to disease) or a 4 (outcome was *possibly* due to treatment and/or suboptimal medical care (50/50 chance) and was *more likely* due to treatment and/or medical management than disease) response options on the 6-point scale. However, raters recognized that the inclusion of both options was important, as in ‘real world’ there are situations in which is difficult to differentiate between disease progression and treatment/medical management and the two options make raters to consider this difference. Perhaps, testing a modify version of this scale, compressing the scale to a 5-point scale (merging options 3 and 4) could be consider. Any such adjustment of the 6-point scale to a 5-point scale would require some retesting of the performance of the revised scale in inter-observer agreement.

Adverse events are an important patient safety issue and have become a focal point for research over the past 30 years. Despite the increased awareness surrounding AEs, recent studies have pointed to the disappointing numbers in AEs trends: rates have not been decreasing significantly [50, 51]. A caveat to these findings, however, is that standardized methodologies for the detection and measurement of AEs need further development and study. Our proposed framework can offer a standardized method that incorporates patients’ views in the assessment of adverse events. Future studies can focus on the validity and reliability of this framework. Widespread adoption of this tool can create a platform for future researchers to compare AE statistic following patient safety interventions.

## Conclusion

The framework presented here is an approach to determining AEs that can be used as a template by clinicians, researchers and health care institutions. The data collected using this framework could help health care systems in their efforts to improve patient safety.

## Abbreviations

AE(s), adverse event(s); AO, adverse outcome; PRAO, patient –reported adverse outcome; RCT, randomized clinical trial.

## Additional files

Additional file 1: Patient Reported Adverse Outcome Questionnaire. (DOC 25 kb) Additional file 2: Health Services Utilization Questionnaire. (DOCX 56 kb) Additional file 3 Patient Reported Outcome (PRAO) Assessment. (DOCX 53 kb) Additional file 4: Type of Adverse Event. (DOCX 61 kb) Additional file 5: Cases reported. (DOCX 989 kb)
