# Erratum to: A framework to assess patient-reported adverse outcomes arising during hospitalization

**DOI:** 10.1186/s12913-017-2392-z

**Published:** 2017-08-16

**Authors:** B. Okoniewska, M. J. Santana, J. Holroyd-Leduc, W. Flemons, M. O’Beirne, D. White, W. Ocampo, W. A. Ghali, A. J. Forster

**Affiliations:** 1 W21C Research and Innovation Centre, G-01- TRW Building, 3280 Hospital Drive, NW, Calgary, AB T2N 4Z6 Canada; 2 Department of Community Health Sciences, O’Brien Institute for Public Health, Cumming School of Medicine, University of Calgary, 3rd Floor, 3-36E, TRW Building, 3280 Hospital Drive, NW, Calgary, AB T2N 4Z6 Canada; 3 Department of Medicine, Cumming School of Medicine, University of Calgary, 1403 29 St NW, Calgary, AB T2N 2 T9 Canada; 4 Family Medicine and Primary Care Research Office, University of Calgary, G012, Health Sciences Centre, 3330 Hospital Drive NW, Calgary, Alberta T2N 4 N1 Canada; 5 Faculty of Nursing, University of Calgary, 2500 University Drive NW, Calgary, AB T2N 1 N4 Canada; 6 Department of Medicine, University of Ottawa, Civic Campus 1053 Carling Avenue, Box 684, Ottawa, ON K1Y 4E9 Canada

## Erratum

Following publication of the original article [1] the authors noticed that only the authors’ first names were listed (except for the last two). The correct authors should be as follows: Okoniewska B, Santana MJ, Holroyd-Leduc J, Flemons W, O'Beirne M, White D, Ocampo W, Ghali WA, Forster AJ.
