# Supplementary material for: A framework to assess patient-reported adverse outcomes arising during hospitalization
Source: BMC Health Serv Res. 2016 Aug 5;16:357. doi: 10.1186/s12913-016-1526-z (PMC4974809; doi:10.1186/s12913-016-1526-z)
Supplement: Additional file 1: — Patient Reported Adverse Outcome Questionnaire. (DOC 25 kb) [file 12913_2016_1526_MOESM1_ESM.doc]

**Appendix 1**

**Patient Reported Adverse Outcome Questionnaire**

1. Since your discharge from the hospital have you experienced a:
   1. New health condition
   2. Worsening health condition
   3. Neither

**If the patient experienced either a new health condition or a worsening condition, proceed to question 2. A different questionnaire should be filled out for each individual problem.**

1. Describe each new health problem
2. When did this problem/condition start (calendar date)?
3. How long did it last?
   1. Less than 1 day
   2. Less than 1 week
   3. Less than 2 weeks
   4. 2 weeks or more
   5. Still Occurring
4. How much has the health problem/condition bothered you? (1 = did not bother them; 10 = bothered them a great deal)
5. How much did this health problem limit your usual physical activity (such as walking or climbing stairs)?
   1. Not at all
   2. Very little
   3. Somewhat
   4. Quite a lot
   5. Could not do
6. Did you do anything about this problem?
   1. Yes
   2. No
7. Describe what was done about the problem
   1. Spoke or visited a doctor
   2. Spoke or visited a pharmacist
   3. Called HealthLink
   4. Went to the Emergency Department
   5. Decided what to do on your own
   6. Other
