# Supplementary material for: A framework to assess patient-reported adverse outcomes arising during hospitalization
Source: BMC Health Serv Res. 2016 Aug 5;16:357. doi: 10.1186/s12913-016-1526-z (PMC4974809; doi:10.1186/s12913-016-1526-z)
Supplement: Additional file 2: — Health Services Utilization Questionnaire. (DOCX 56 kb) [file 12913_2016_1526_MOESM2_ESM.docx]

**Appendix 2**

**Health Services Utilization Questionnaire**

1. Since your discharge have you visited a doctors office?
   1. Yes
   2. No

**If No, proceed to question 5. If Yes, proceed to question 2**

1. What was the reason for the visit?
2. When was the visit? (calendar date)
3. What was the result of the visit?
4. Since your discharge have you gone to the Emergency Department?
   1. Yes
   2. No

**If No, proceed to question 9. If Yes, proceed to question 6**

1. What was the reason for the visit?
2. When was the visit? (calendar date)
3. What was the result of the visit?
4. Since you left the hospital have you been readmitted to the hospital?
   1. Yes
   2. No

**If No, this is the end of the questionaire. If Yes, proceed to question 10.**

1. What was the reason for the visit?
2. When were you admitted? (calendar date)
3. When were you discharged? (calendar date)
4. What was the result of the visit?
