# Supplementary material for: A framework to assess patient-reported adverse outcomes arising during hospitalization
Source: BMC Health Serv Res. 2016 Aug 5;16:357. doi: 10.1186/s12913-016-1526-z (PMC4974809; doi:10.1186/s12913-016-1526-z)
Supplement: Additional file 3 — Patient Reported Outcome (PRAO) Assessment. (DOCX 53 kb) [file 12913_2016_1526_MOESM3_ESM.docx]

**Appendix 3**

**Patient Reported Outcome (PRAO) Assessment**

1. Determine the onset of the symptom
   1. Before health encounter/intervention
   2. During health encounter/intervention
   3. After health encounter/intervention
2. What alleviated the symptom?

______________________________________________________________________

1. Duration of the symptom
   1. Less than 1 day
   2. Less than 1 week
   3. Less than 2 weeks
   4. 2 weeks or more
   5. Still Occurring
2. What health services were utilized? (circle one)
   1. None
   2. Additional visit to a physician, that is, one that was arranged specifically for the particular symptom, not follow up visits made prior to discharge
   3. Additional visit for laboratory testing in addition to a physician visit
   4. Visit to the Emergency Department
   5. Readmission to hospital
3. What was the effect of the PRAO on the patient’s health (circle one)?
   1. Laboratory Abnormality only
   2. Symptoms only
   3. Nonpermanent Disability – to be considered an nonpermanent disability instead of several days of symptoms, there must be evidence that symptoms interfered with patients’ activities of daily living
   4. Permanent Disability
   5. Disability but cannot determine permanence
   6. Death
