# Supplementary material for: A framework to assess patient-reported adverse outcomes arising during hospitalization
Source: BMC Health Serv Res. 2016 Aug 5;16:357. doi: 10.1186/s12913-016-1526-z (PMC4974809; doi:10.1186/s12913-016-1526-z)
Supplement: Additional file 4: — Type of Adverse Event. (DOCX 61 kb) [file 12913_2016_1526_MOESM4_ESM.docx]

**Appendix 4**

**Patient ID # ______________**

After reading the case report of the patient, please answer the following questions:

1. Was the patient reported adverse outcome due to medical care?
   1. Yes
   2. No

***If you replied Yes, please proceed to the next question.***

1. Determine the type of Adverse Event:
   1. Adverse Drug Event
   2. Procedure-related injury
   3. Nosocomial infection
   4. Care Related Fall
   5. Therapeutic Error
   6. Diagnostic Error (an indicated test was not ordered ot a significant test result was misinterpreted)
   7. Other
2. Determine whether the adverse event was one of the following:
   1. **Ameliorable** – an injury whose severity could have been *substantially reduced* if different actions or procedures had been performed or followed (unavoidable injuries but severity could have been decreased).
   2. **Preventable** – an injury that could have been *avoided*, that is, an injury judged to probably be the result of an error or a system design flaw.
   3. **Neither**

Rate your confidence that the new problem or a worsening condition was caused by an error or health system flaw. An error is defined as a failure to achieve a desired objective through the failure to execute a plan correctly, through the implementation of an incorrect plan, or through omission.

A rating of *5 or 6* indicates that the injury was probably or definitely caused by management and is *considered a preventable adverse event****.*** Circle the most appropriate choice:

- 1. No evidence that outcome was due to treatment and/or suboptimal medical care
  2. Little evidence that outcome was due to treatment and/or suboptimal medical care
  3. Outcome was possibly due to treatment and/or suboptimal medical care (50/50 chance) but was more likely due to disease
  4. Outcome was possibly due to treatment and/or suboptimal medical care (50/50 chance) and was more likely due to treatment and/or medical care than disease
  5. Outcome was probably due to treatment and/or suboptimal medical care
  6. Outcome was definitely due to treatment and/or suboptimal medical care
