# Supplementary material for: A framework to assess patient-reported adverse outcomes arising during hospitalization
Source: BMC Health Serv Res. 2016 Aug 5;16:357. doi: 10.1186/s12913-016-1526-z (PMC4974809; doi:10.1186/s12913-016-1526-z)
Supplement: Additional file 5: — Cases reported. (DOCX 989 kb) [file 12913_2016_1526_MOESM5_ESM.docx]

**Appendix 5**

**Appendix 5a – Case 1494**

Case number 1494 (Appendix 6a) is an example where both raters unanimous agreed that the PRAO was due to medical care and it was classified as an ameliorable adverse drug event. On the 6-point scale, both reviewers rated the case as a 5.

**Patient ID #1494**

**New Problem at 1 month as described by patient**

*70 yo Male*

| History | Pt reported having a negative side effect to a drug, lomotil. He was prescribed this at the end of his hospital stay. Pt started taking lomotil after leaving the hospital. Pt said the side effects started around 2 weeks after starting the medication, post discharge and described the side effects as making him dizzy so that he could not stand up and get around. |
| --- | --- |
| Onset of symptoms | June 24 |
| Length of problem | Less than 2 weeks |
| Severity of Problem (scale of 1-10) | 5 |
| How much did the problem limit the pt’s usual activities? (scale 1-5) | 4 |
| What was done about health problem | Pt did not see a doctor but stopped taking the medication. Side effects diminished and then stopped about a week after stopping medication. |

**Patient’s Original Stay in Hospital (Discharge Summary)**

*70 yo Male*

| LOS | 6 days |
| --- | --- |
| Most Responsible Diagnosis | Decreased LOC |
| Secondary Diagnosis | -- |
| Discharge Medications | - Pantoprazole EC tab 40 mg PO bid before meals - Psyllium mucilloid powder 1 packet PO bid - Diphenoxylate/atropine tab (Lomotil) 1 tab PO tid - Sulfamethoxazole/trimethoprim DS tab (Septra OS tab) 1 tab PO daily, Stop After 3 Days |
| Additional Notes | -- |

**Brief Discharge Summary**

*Presentation*

- Pt was admitted to the FMC on June 8th after a bout of green/black emesis.
- This event was precipitated by a third spontaneous bowel obstruction this year.

*Past Medical History*

- Crohn's disease, which was treated with a proctransfer-of-careollectomy and ileostomy in 1995, and BPH, which was treated with a TURP in 2005.

*Issues*

- Pt was investigated by GI for active Crohn’s disease, and the endoscope did not reveal an active disease
- It did reveal a stricture at the stoma site.
- On his fourth day of admission, the pt was retaining urine most likely due to a traumatic catheter insertion on his admission.
- His urinary retention was treated with a foley catheter, which was removed under the instructions of the Urologist on-call, Dr. W.

**Brief Discharge Instructions**

Pt is to follow up with his gastroenterologist, Dr. K, and with Dr. W in the case that he retains urine on d/c

**Reviewer 1’s Original Rating of Case Study**

**
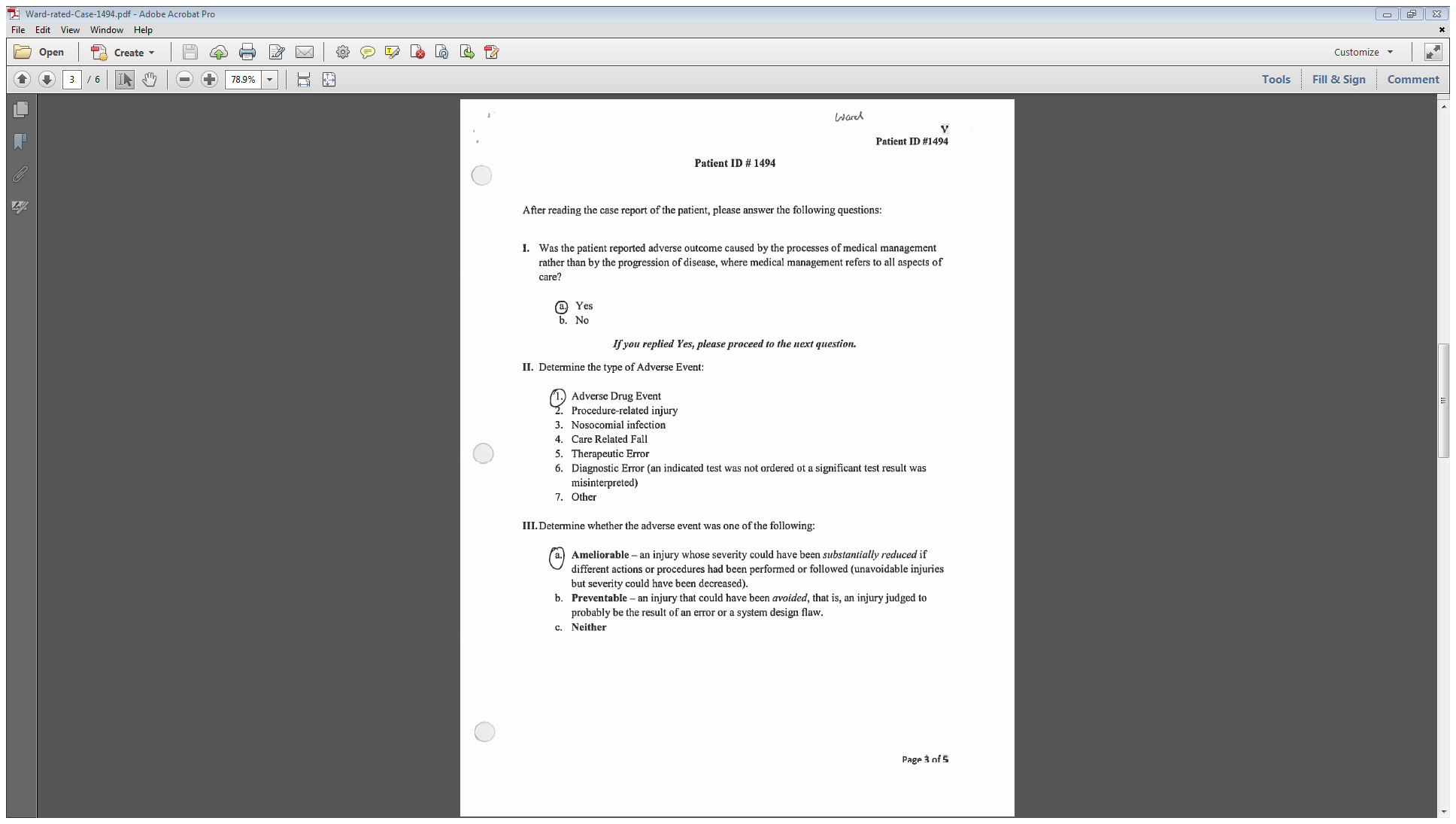
**

**
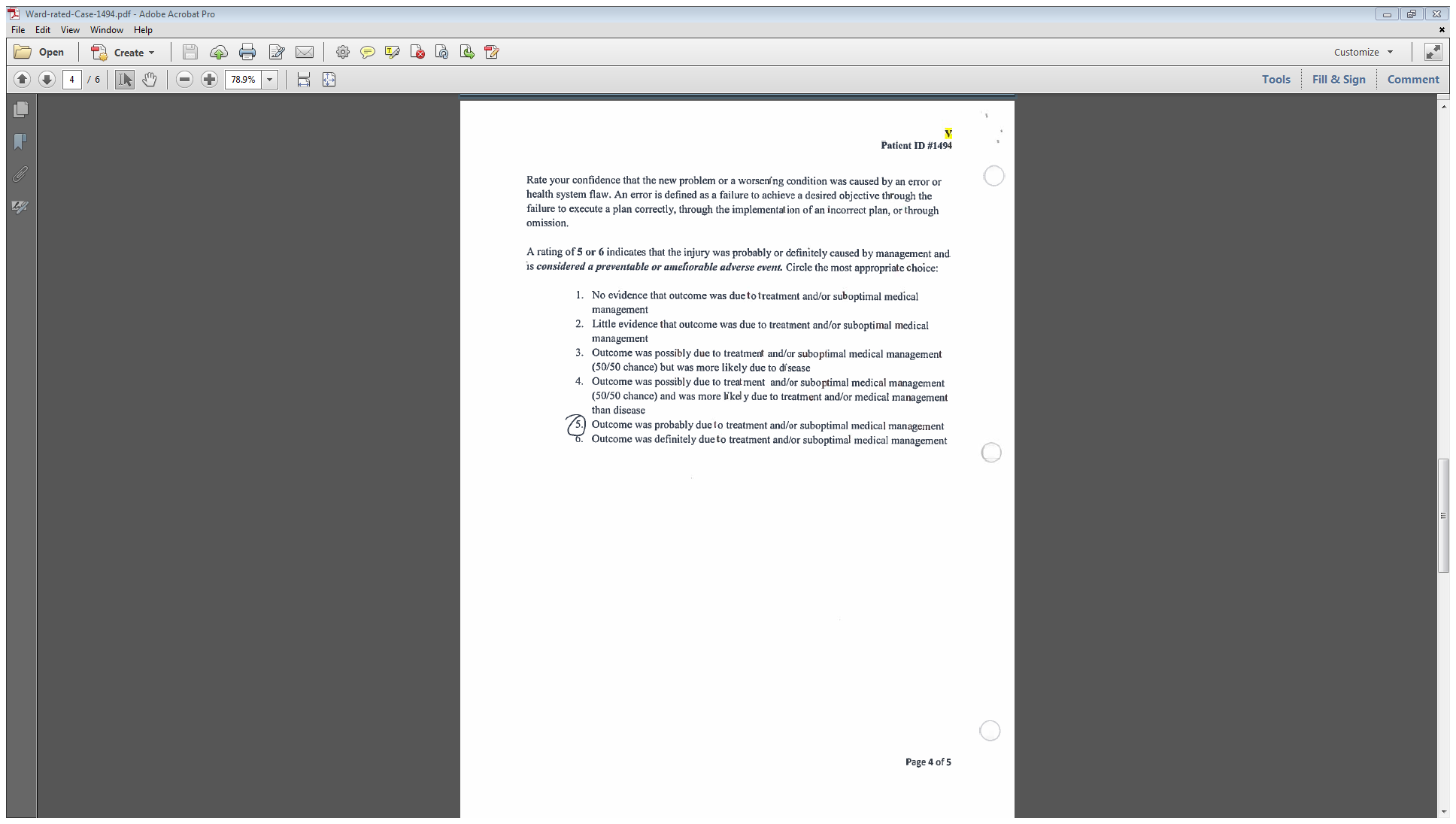
**

**Reviewer 3’s Original Rating of Case Study**

**
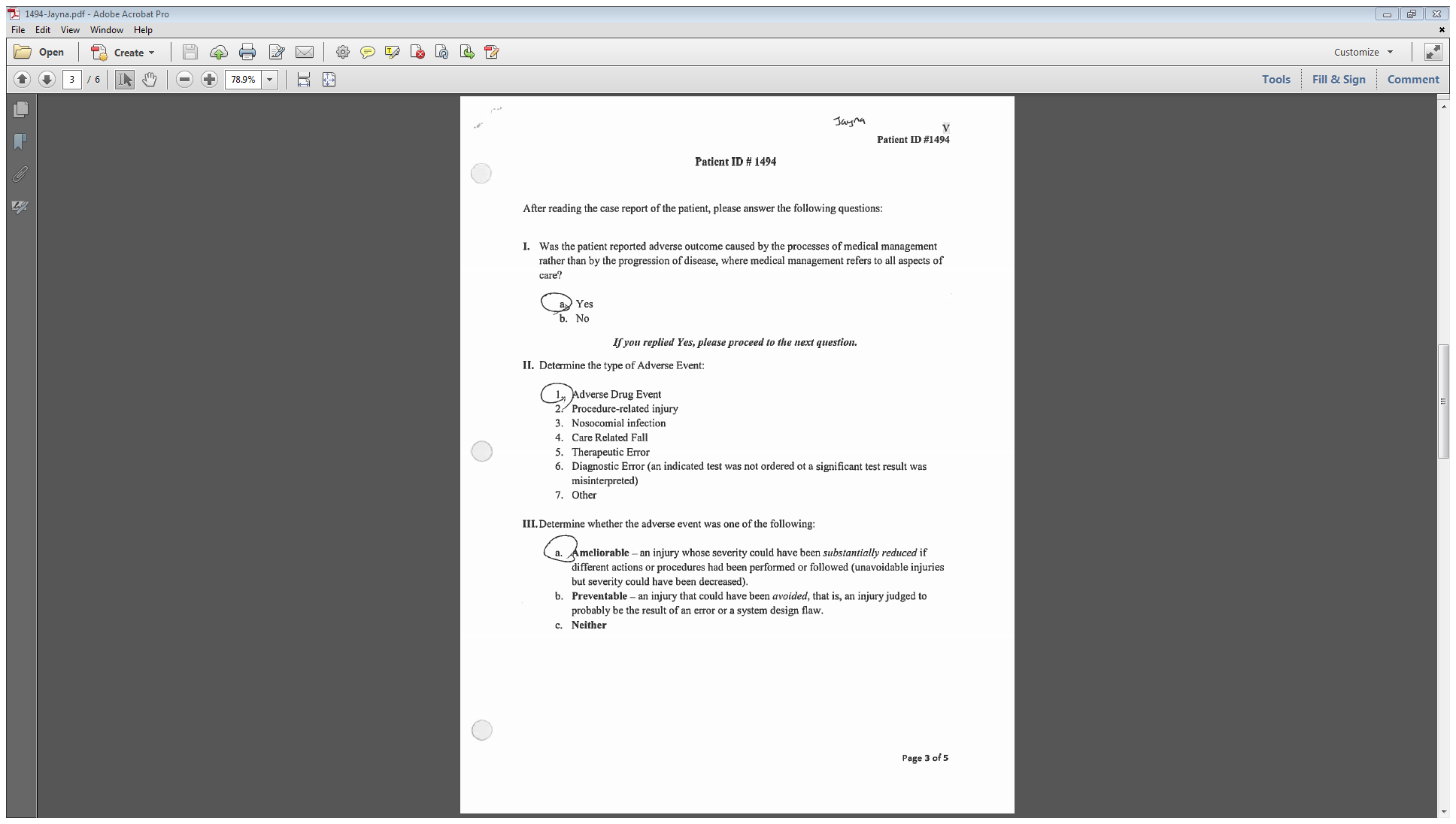
**

**
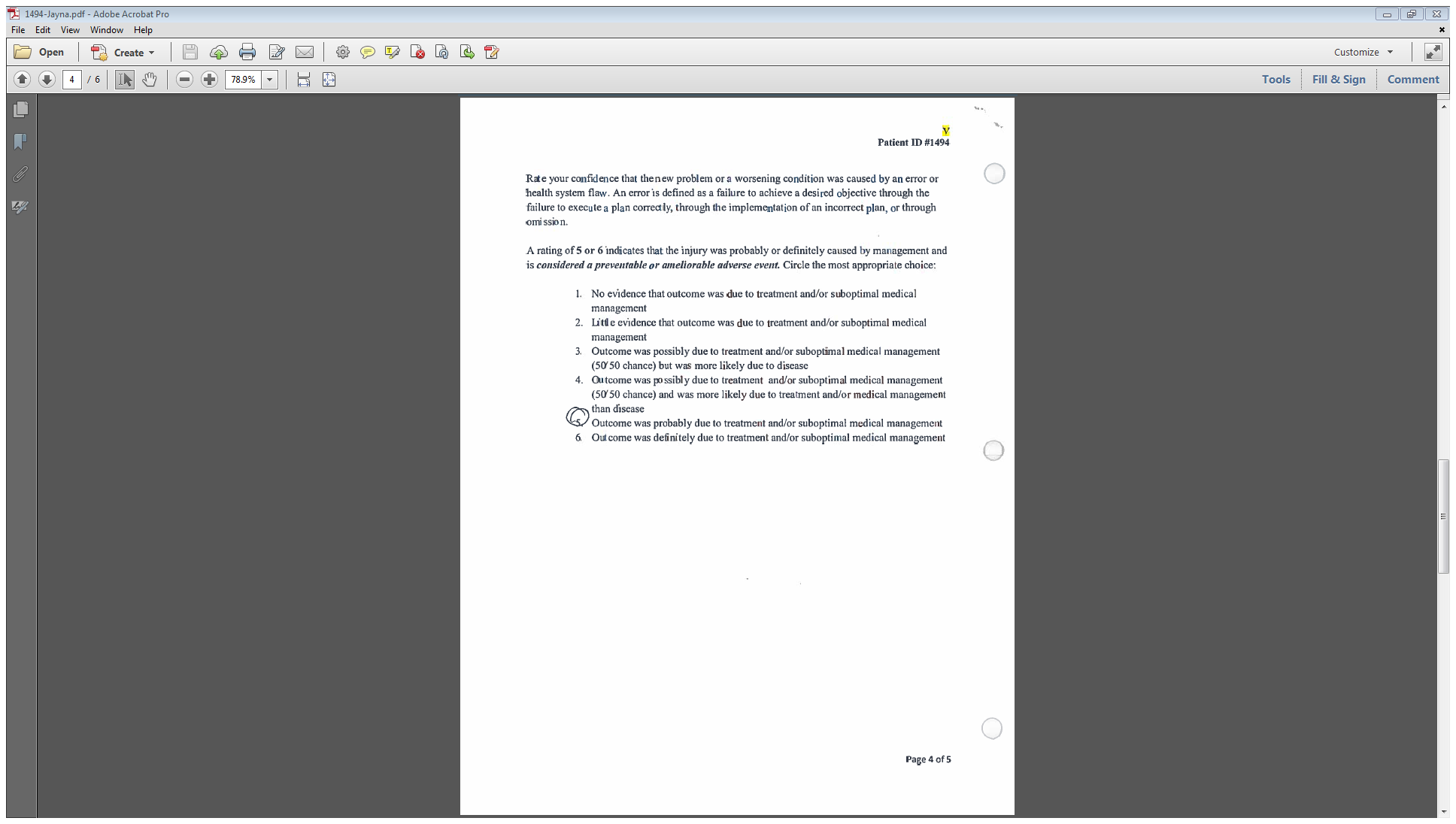
**

**Appendix 5b – Case 1211**

Case number 1211 (Appendix 6b) illustrates a case in which both reviewers agreed that the PRAO was due to medical care, neither of them thought that the AE was caused by an error or health system flaw, rating the case 3 on the 6-point scale. Both reviewers also agreed that *neither* an ameliorable or preventable AE occurred.

**Patient ID #1211**

**New Problem at 1 month as described by patient**

*76 yo Male*

| History | Pt. had a bladder infection while in the hospital. Pt was d/c with medication given by mouth (Pt's daughter was unsure what it was). After medication was completed the pain upon urination and discomfort returned. |
| --- | --- |
| Onset of symptoms | About one week after d/c |
| Length of problem | Still occurring |
| Severity of Problem (scale of 1-10) | 7 |
| How much did the problem limit the pt’s usual activities? (scale 1-5) | 3 |
| What was done about health problem | Pt's daughter made a Dr appointment for tomorrow, April 10th for him to be seen, and expects that he will be given additional medication to assist with what she suspects as an ongoing infection and mild fever. |

**Patient’s Original Stay in Hospital (Discharge Summary)**

*76 yo Male*

| LOS | 5 days |
| --- | --- |
| Most Responsible Diagnosis | Pyelonephritis/sepsis |
| Secondary Diagnosis | - AKI - Rhabdomyolysis - Tropinitis, secondary to rhabdo and demand query. Needs stress test arranged |
| Discharge Medications | - Acetaminophen tab 650 mg PO qid - Ciprofloxacin tab 500 mg PO bid, stop after 6 days - Gliclazide tab 160 mg PO bid with meals - Hydrochlorothiazide tab 25 mg PO daily - Losartan tab 100 mg PO daily - Metformin tab 1000 mg PO every 1 day: 08:00, 17:00 (breakfast and supper) - Metformin tab 500 mg PO every 1 day: 1200 with lunch - Rabeprazole EC (Pariet) tablet 20 mg PO daily for d/c. Pharmacy not to send. Pt is stabilized on non-formulary medication |
| Additional Notes | Ex-smoker: 40-50 packs a year. Quit about 20 years ago |

**Brief Discharge Summary**

*Presentation*

- Pt was found unresponsive at home by family members after a 1 week history of feeling unwell with myalgias, malaise and urinary symptoms
- His family thought he was potentially unresponsive for several hours

*Past Medical History*

- Type 2 DM: diagnosed more than 10 years, non-insulin dependent, mild proteinurea
- Hypertension

*Labs/Tests*

- Urine and blood cultures were found to be positive for Gram negative coliforms (Klebsiella pneumonia), and he was diagnosed with Gram negative urosepsis, rhabdomyolysis and elevated troponin. This was treated with ceftriaxone IV and fluids
- Creatine was 128, CK was 4215 and urine positive for myoglobin
- Kidneys, ureters and bladder ultrasound were unremarkable
- His lack of cardiac symptoms and non-progressive EKGs suggested his troponin elevation was due to transient renal failure from rhabdomyolysis

*Issues*

- Throughout his hospital stay, WBC trended down to normal limits, and other abnormal lab values trended down
- His blood sugars remained labile, and often trended into the 10-13 range
- He was stepped down from IV ceftriaxone to PO ciprofloxacin 500 mg bid without issue, and voided once the foley was removed

*Discharge*

- Creatinine was normal
- EKG showed no evidence of myocardial ischemia

**Brief Discharge Instructions**

1. Urosepsis

- Pt to continue on ciprofloxacin 500 mg PO bid for total antibiotic course of 10 days
- No evidence of pyelonephritis on ultrasound of kidneys, ureters and bladder

1. Diabetes

- Insulin sliding discontinued on the day of d/c (previously requiring short acting insulin 6 units during lunch)
- Pt’s home oral agents restarted March 12, but his medication does and actual use at home will need to be reassessed

1. Troponin elevation

- Likely demand ischemia secondary to urosepsis, acute renal dysfunction and rhabdomyolysis
- When appropriate, his family dr to arrange a stress test (e.g. exercise stress test) for cardiac risk stratification

Family physician f/u required by March 18 and the appointment will be arranged

**Reviewer 1’s Original Rating of Case Study**

**
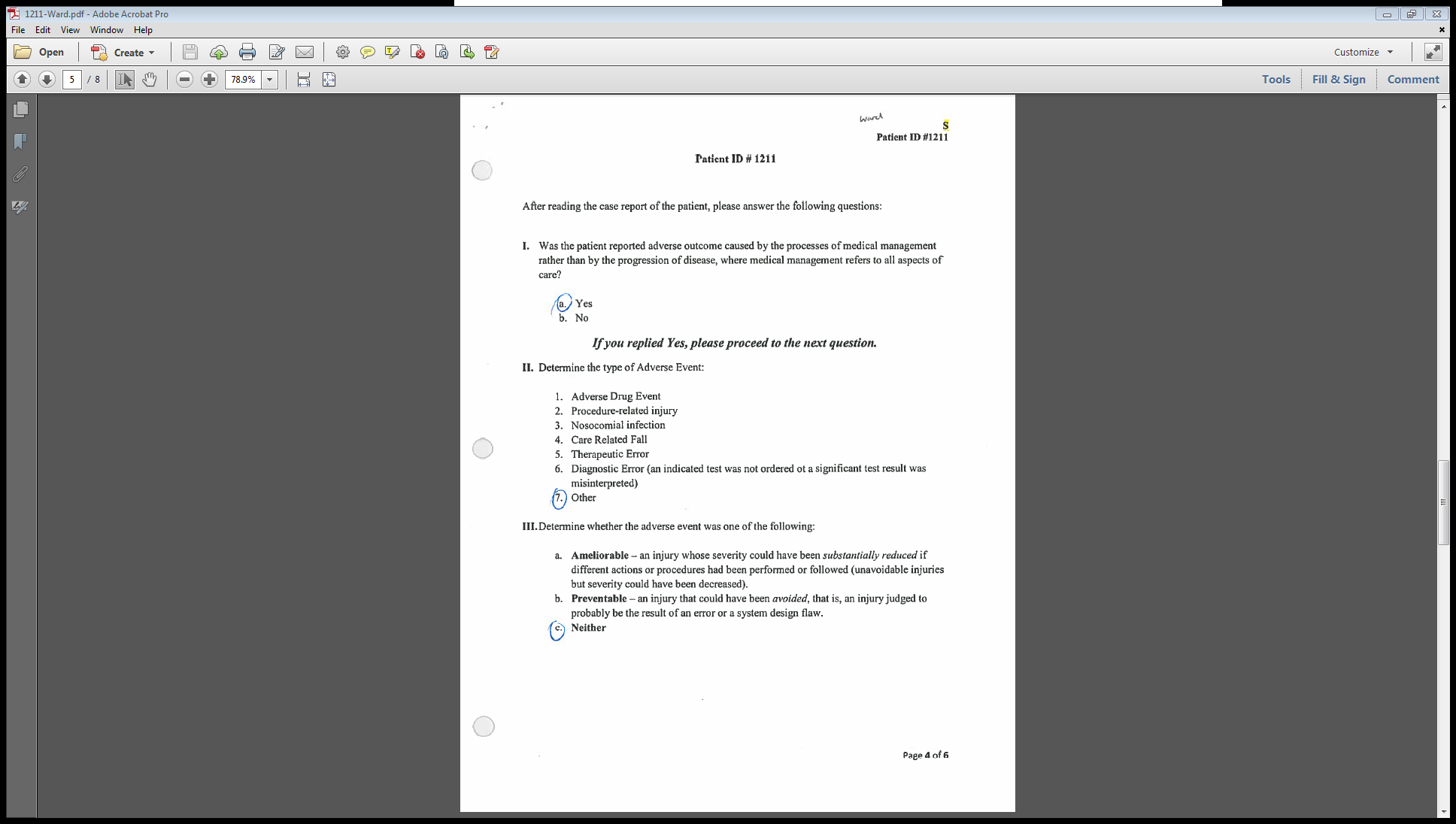
**

**
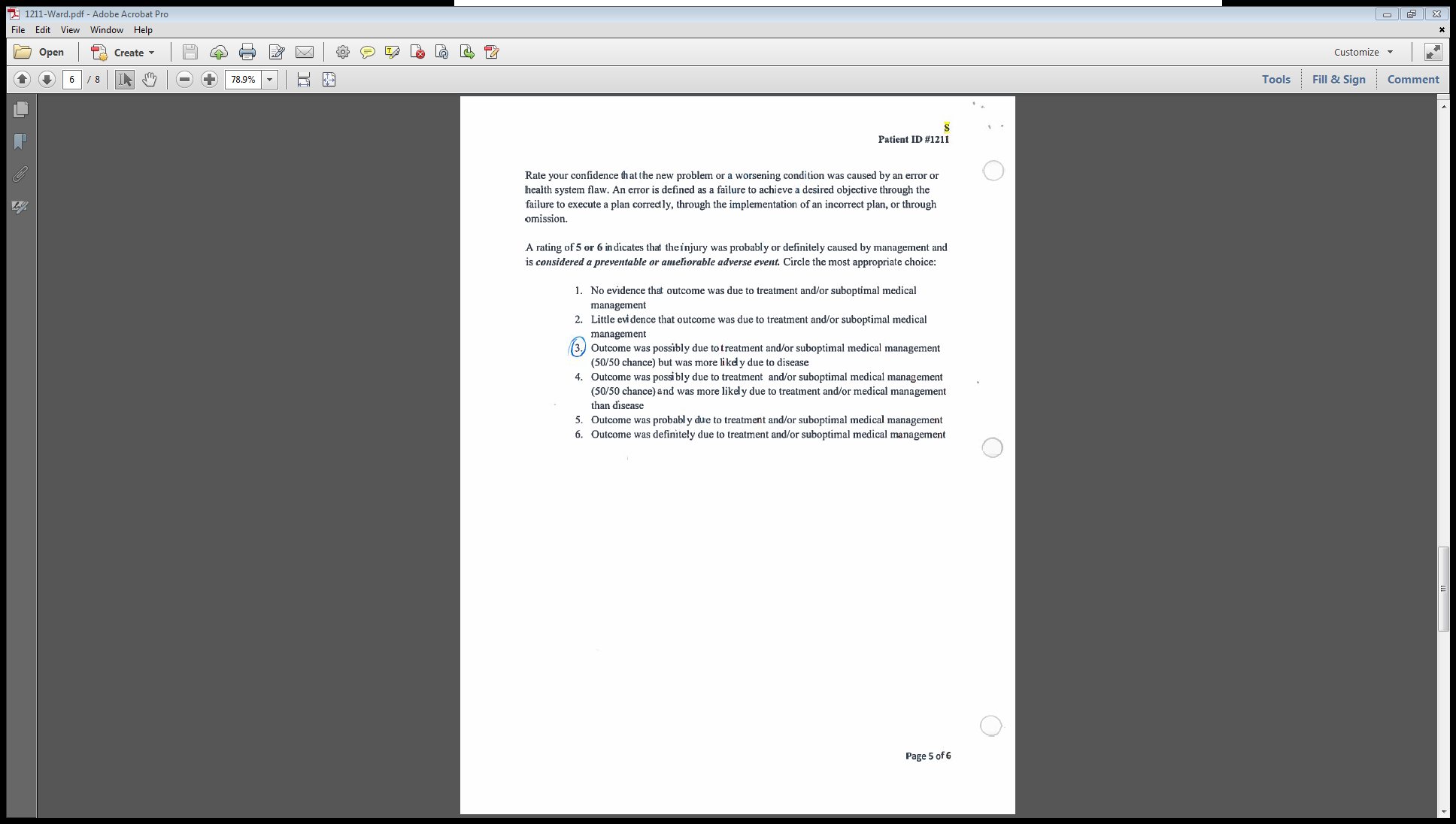
**

**Reviewer 2’s Original Rating of Case Study**

**
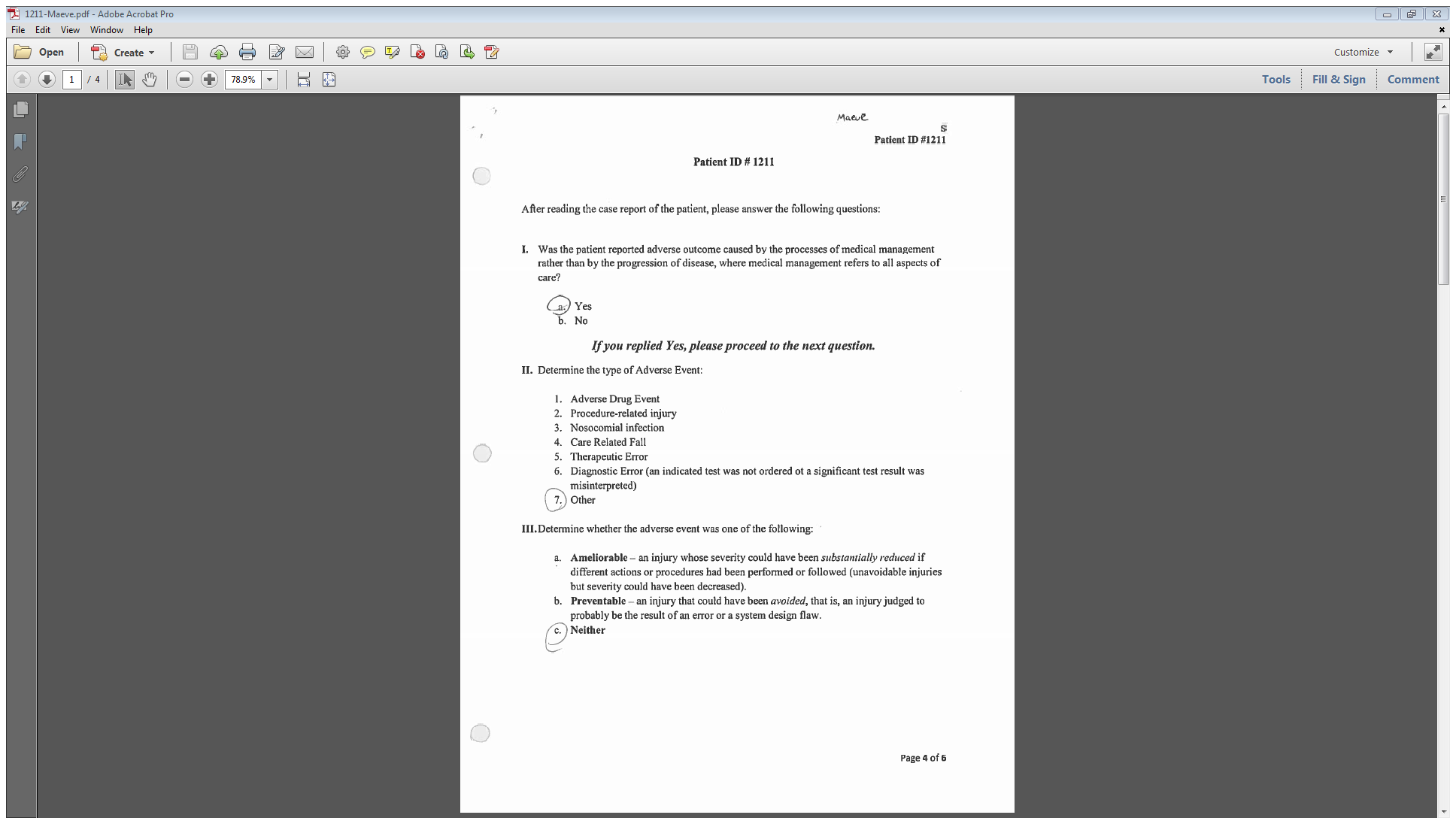
**

**
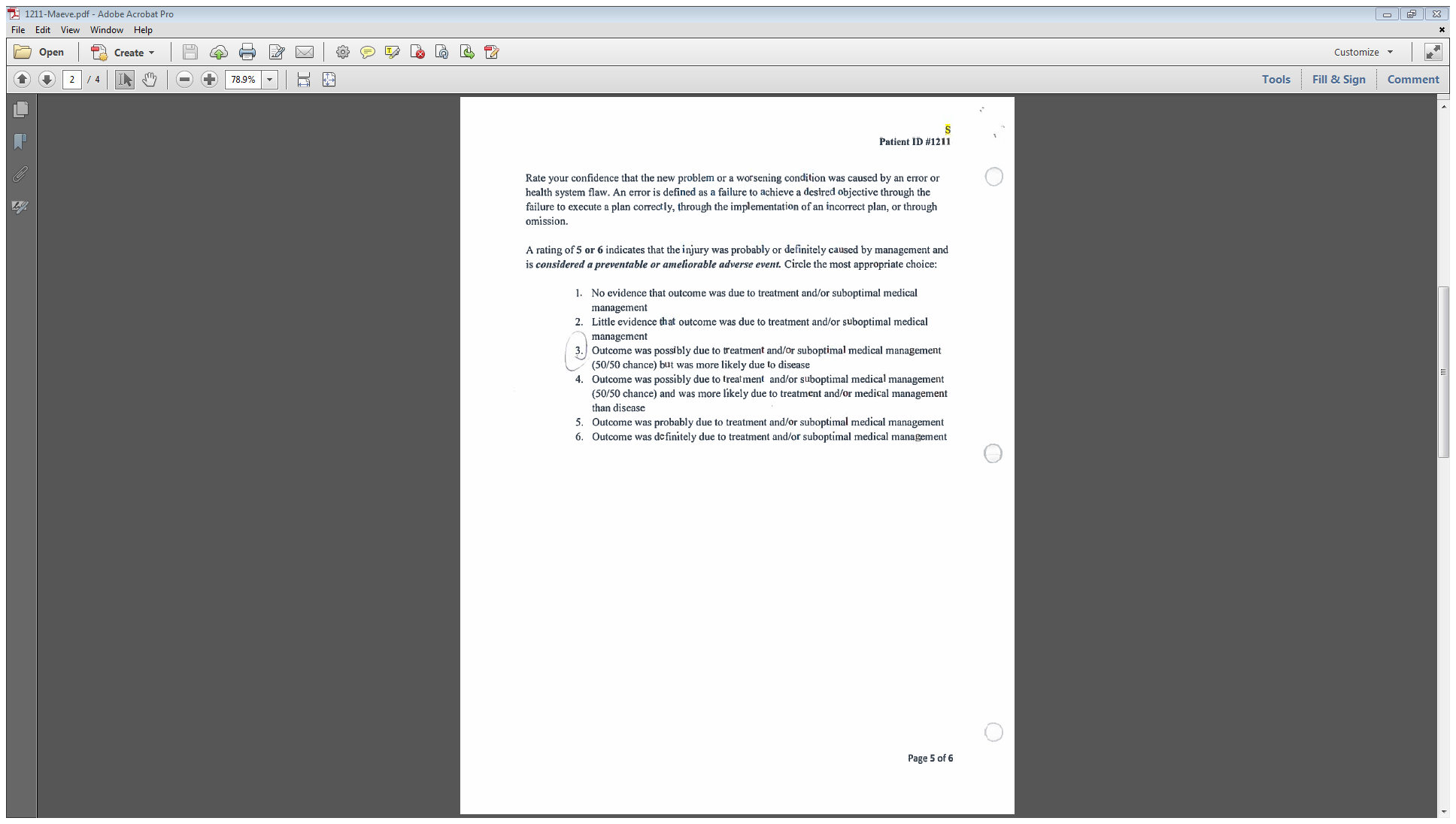
**

**Appendix 5c – Case 947**

Case number 947 (Appendix 6c) is an example of disagreement – one reviewer thought the PRAO was due to medical care, and went on to classify it as preventable adverse drug event, and a 5 on the 6-point scale. The other reviewer did not think the PRAO was due to medical care. Both reviewers were asked to meet, review and re-rate the case. After this process, they agreed that the PRAO was due to medical care, although it was not due to an error or health system flaw, rating it 2 out of 6 on the 6-point scale.

**Patient ID #947**

**New Problem at 1 month as described by patient**

*39 yo Male*

| History | Pt was experiencing all kinds of pain within a few days post discharge. Dizziness, pain in the side, weight loss and numbness with his hands. Has gone to the emergency room x3 since discharge. At discharge was told that a hematologist was to contact him within a week post d/c - was never contacted. Has attempted to call the clinic on numerous occasions and was not able to get an appointment. |
| --- | --- |
| Onset of symptoms | A few days post d/c |
| Length of problem | Still Occurring |
| Severity of Problem (scale of 1-10) | 10 |
| How much did the problem limit the pt’s usual activities? (scale 1-5) | 4 |
| What was done about health problem | Pt went to his GP who sent him to the ED three times. In the ED he was kept overnight for observation |

**Patient’s Original Stay in Hospital (Discharge Summary)**

*39 yo Male*

| LOS | 9 days |
| --- | --- |
| Most Responsible Diagnosis | Alcoholic hepatitis, alcoholic cirrhosis, lower GI bleed secondary to hemorrhoids. |
| Secondary Diagnosis | - Hypertension - Hyperthyroidism - Alcohol abuse |
| Discharge Medications | New medications:   - .Pentoxitylline 400 mg p.o. t.i.d. to stop January 1st (last - dose) - Nadolol 40 mg p.o. daily - Spironolactone 100 mg p.o. daily - Lasix 40 mg p.o. daily - Centrum 1 tablet p.o. daily - Ferrous gluconate 300 mg p.o. b.i.d - Pantoprazole 40 mg p.o. daily - Calcium carbonate 1250 mg p.o. daily   Home medications to be continued:   - Levothyroxine 75 meg p.o. daily - Zopiclone 7.5 mg p.o. daily.   Discontinued medications:   - Atacand 16/12.5 mg. |
| Additional Notes |  |

**Brief Discharge Summary**

*Presentation*

- Presented with a 5-week history of increased abdominal girth, jaundice of his skin, as well as increased fatigue and general unwellness.
- Admitted for workup of his hepatitis and cirrhosis, as well as his bright red blood per rectum

Issues

1. Alcoholic hepatitis

- Increase in his liver enzymes on admission specifically his bilirubin was 146, GTT, lipase 84, ALT 40 and on discharge his bilirubin was 92. His GTT was 87 and his ALT was 25.
- Maddrey score of 44
- The decision was made to begin him on a 28- day course of pentoxifylline 400 mg p.o. t.i.d. His course is to end on January 31, 2013

1. Alcoholic cirrhosis

- Underwent the basic workup for chronic liver disease in order to rule out any competing diagnosis.
- His workup including viral hepatitis serology as well as alpha 1 antitrypsin and ceruloplasmin came back negative
- Diagnosis of alcoholic cirrhosis was made.
- Underwent an ultrasound to look more carefully at the liver and the ultrasound revealed cirrhosis and portal hypertension.
- He had a negative hepatoma screen.
- Hepatic venous and portal venous vasculature was normal.
- For his cirrhosis, we initiated him on a combination of diuretics including Lasix and spironolactone.
- On discharge, he was on spironolactone 100 mg p.o. daily and Lasix 40 mg p.o. daily, as he has been quite volume overloaded.
  - These diuretics can be reviewed by his family doctor and it is reasonable to reduce the spironolactone down to 50 mg p.o. daily and the Lasix down to 20 mg p.o. daily if you feel that his ascites and leg edema has reduced.
- Child-Pugh scoring, pt referred to hepatology to be followed up as an outpatient and to be assessed for appropriateness for placement on the liver transplant list. The referral form for the hepatology central intake has been sent and they should be in contact with the patient directly in the weeks to follow

1. Lower GI bleed

- Presented with several week history of bright red blood per rectum, streaking of the stool.
- GI consult
- Underwent an EGO
  - Revealed some gastritis but no source of bleeding.
- Colonoscopy
  - Revealed hemorrhoids as a likely cause of his bleeding per rectum.
  - Reasonable to apply Anusol cream for symptomatic relief.
  - We are treating these hemorrhoids similar to rectal varices in the setting of cirrhosis, therefore we have initiated Nadolol 40 mg p.o. daily to decompress his hemorrhoids.
- He has continued to have minor bleeds during his hospital stay.
- Hemoglobin has remained stable prior to discharge.
- Wade will not need any further follow up with the
- GI doctor upon leaving the hospital unless there is reason to re-scope him such as ongoing bleeding requiring ongoing medical attention

1. Volume overload

- Wade had an acute decompensation on December 7th and 8th which lead to a vast increase in abdominal ascites that he had as well as the amount of peripheral edema that he had although this is most likely due to his cirrhosis.
- Wanted to rule out the 2 competing primary causes of volume overload, namely heart failure and nephrotic syndrome.
- Ordered a urine microalbumin to creatinine ratio as well as an echo.
- These tests have been done prior to his discharge from the hospital
  - they will require to be followed up by his family doctor upon discharge

1. Alcohol abuse

- Seen by the Addiction Service at the Foothills Medical Centre.
- Provided him with several resources in the community that could aid in sensation of alcohol.
- Cadency for being added to the liver transplant list:
  - He will need to be abstinent from alcohol for at least 6 months and as well, he will need to complete the ADDAC program prior to hepatology considering adding him to the transplant list.

**Brief Discharge Instructions**

- The patient should see his GP within 1 week's time in order to review his electrolytes, his creatinine as well as the dose of his diuretics (Lasix plus spironolactone). His GP should also monitor his volume status and his respiratory effort
- Pt should continue to abstain from alcohol indefinitely but most definitely for the next 6 months in order to be considered for the liver transplant list.
- Needs to undergo ADDAC addictions counseling. He is aware of both of these things
- Pt has been referred to the hepatology service through central intake and they will be in contact in regards to an appointment date.

**Reviewer 1’s Original Rating of Case Study**
